# Screening for Tuberculosis in Migrants: A Survey by the Global Tuberculosis Network

**DOI:** 10.3390/antibiotics10111355

**Published:** 2021-11-05

**Authors:** Lia D’Ambrosio, Rosella Centis, Claudia C. Dobler, Simon Tiberi, Alberto Matteelli, Justin Denholm, Dominik Zenner, Seif Al-Abri, Fatma Alyaquobi, Marcos Abdo Arbex, Evgeny Belilovskiy, François-Xavier Blanc, Sergey Borisov, Anna Cristina C. Carvalho, Jeremiah Muhwa Chakaya, Nicola Cocco, Luigi Ruffo Codecasa, Margareth Pretti Dalcolmo, Keertan Dheda, Anh Tuan Dinh-Xuan, Susanna R. Esposito, José-María García-García, Yang Li, Selene Manga, Valentina Marchese, Marcela Muñoz Torrico, Emanuele Pontali, Adrián Rendon, Denise Rossato Silva, Rupak Singla, Ivan Solovic, Giovanni Sotgiu, Martin van den Boom, Nguyen Viet Nhung, Jean-Pierre Zellweger, Giovanni Battista Migliori

**Affiliations:** 1 Public Health Consulting Group, 6900 Lugano, Switzerland; liadambrosio59@gmail.com; 2 Servizio di Epidemiologia, Clinica delle Malattie Respiratorie, Istituti Clinici Scientifici Maugeri IRCCS, 21049 Tradate, Italy; rosella.centis@icsmaugeri.it; 3 The George Institute for Global Health, University of New South Wales, Sydney, NSW 2042, Australia; c.dobler@unsw.edu.au; 4 Department of Respiratory and Sleep Medicine, Liverpool Hospital, Sydney, NSW 2107, Australia; 5 Department of Infection, Royal London Hospital, Barts Health NHS Trust, London E1 1FR, UK; s.tiberi@qmul.ac.uk; 6 Blizard Institute, Queen Mary University of London, London E1 2AT, UK; 7 Division of Infectious and Tropical Diseases, Spedali Civili University Hospital, 25123 Brescia, Italy; alberto.matteelli@unibs.it (A.M.); v.marchese@unibs.it (V.M.); 8 WHO Collaborating Centre for TB/HIV Collaborative Activities and for TB Elimination Strategy, University of Brescia, 25123 Brescia, Italy; 9 Victorian Tuberculosis Program, Melbourne Health, Melbourne, VIC 3000, Australia; justin.denholm@mh.org.au; 10 Department of Infectious Diseases, Peter Doherty Institute for Infection and Immunity, University of Melbourne, Melbourne, VIC 3010, Australia; 11 Victorian Infectious Diseases Service, Royal Melbourne Hospital, Melbourne, VIC 3050, Australia; 12 Centre for Global Public Health, Institute for Population Health Sciences, Queen Mary University, London E1 2AB, UK; dominik.zenner@ucl.ac.uk; 13 Directorate General of Disease Surveillance and Control, Ministry of Health, Muscat 100, Oman; salabri@gmail.com (S.A.-A.); fatmayaquobi@gmail.com (F.A.); 14 Nestor Goulart Reis Hospital, Health Secretary São Paulo State, Sao Paulo 14801-320, Brazil; arbexma@techs.com.br; 15 Faculdade de Medicina, Universidade de Araraquara, Sao Paulo 14801-320, Brazil; 16 Moscow Research and Clinical Center for Tuberculosis Control, 107014 Moscow, Russia; belilovsky@gmail.com (E.B.); sebarsik@gmail.com (S.B.); 17 Service de Pneumologie, Centre Hospitalier Universitaire, L’institut du Thorax, F-44093 Nantes, France; xavier.blanc@chu-nantes.fr; 18 Laboratório de Inovações em Terapias, Ensino e Bioprodutos (LITEB), Instituto Oswaldo Cruz, Fundação Oswaldo Cruz, Rio de Janeiro 21040-360, Brazil; carvalhoannacristinac@gmail.com; 19 Department of Medicine, Therapeutics, Dermatology and Psychiatry, Kenyatta University, Nairobi P.O. Box 43844-00100, Kenya; chakaya.jm@gmail.com; 20 Department of Clinical Sciences, Liverpool School of Tropical Medicine, Liverpool L3 5QA, UK; 21 ASST Santi Paolo e Carlo—Medicina Penitenziaria, 21100 Milan, Italy; nicolcocco@gmail.com; 22 TB Reference Centre, Villa Marelli Institute, Niguarda Hospital, 20159 Milan, Italy; luigiruffo.codecasa@ospedaleniguarda.it; 23 Reference Center Hélio Fraga, Fundação Oswaldo Cruz (Fiocruz), Ministry of Health, Rio de Janeiro 21040-360, Brazil; margarethdalcolmo@gmail.com; 24 South African MRC Centre for the Study of Antimicrobial Resistance, University of Cape Town, Cape Town 7701, South Africa; keertan.dheda@uct.ac.za; 25 Centre for Lung Infection and Immunity, Division of Pulmonology, Department of Medicine and UCT Lung Institute, University of Cape Town, Cape Town 7701, South Africa; 26 Department of Infection Biology, Faculty of Infectious and Tropical Diseases, London School of Hygiene and Tropical Medicine, London 400706, UK; 27 Respiratory Physiology Unit, Department of Respiratory Medicine, Cochin Hospital, Université de Paris, 75014 Paris, France; anh-tuan.dinh-xuan@aphp.fr; 28 Paediatric Clinic, Department of Medicine and Surgery, University Hospital, University of Parma, 43126 Parma, Italy; susannamariaroberta.esposito@unipr.it; 29 Tuberculosis Research Programme SEPAR, E-08029 Barcelona, Spain; josemariagarciagarcia@gmail.com; 30 Department of Infectious Diseases, Huashan Hospital, Fudan University, Shanghai 200040, China; losty34217@gmail.com; 31 Ministry of Health, Direccion General de Gestion de Riesgos en y Desastres en Salud, Lima 15072, Peru; seleneperu@yahoo.com.mx; 32 Clínica de Tuberculosis, Instituto Nacional de Enfermedades Respiratorias Ismael Cosio Villegas, Mexico City 14080, Mexico; dra_munoz@hotmail.com; 33 Department of Infectious Diseases, Galliera Hospital, 16128 Genoa, Italy; pontals@yahoo.com; 34 Centro de Investigación, Prevención y Tratamiento de Infecciones Respiratorias CIPTIR, University Hospital of Monterrey UANL (Universidad Autonoma de Nuevo Leon), Monterrey 64000, Mexico; adrianrendon@hotmail.com; 35 Faculdade de Medicina, Universidade Federal do Rio Grande do Sul, Porto Alegre 90035-903, Brazil; denise.rossato@terra.com.br; 36 Department of TB & Respiratory Diseases, National Institute of TB & Respiratory Diseases, Sri Aurobindo Marg, New Delhi 110030, India; drrupaksingla@yahoo.com; 37 National Institute for TB, Vysne Hagy, Catholic University, 05984 Ruzomberok, Slovakia; ivan.solovic@vhagy.sk; 38 Clinical Epidemiology and Medical Statistics Unit, Department of Medical, Surgical and Experimental Sciences, University of Sassari, 07100 Sassari, Italy; gsotgiu@uniss.it; 39 WHO Regional Office for the Eastern Mediterranean Region, Cairo 11571, Egypt; vandenboomm@who.int; 40 National Tuberculosis Programme, Hanoi 100000, Vietnam; vietnhung@yahoo.com; 41 TB Competence Center, Swiss Lung Association, 3030 Berne, Switzerland; zellwegerjp@swissonline.ch

**Keywords:** TB, migration, COVID-19, TB infection management, prevention, infection control, screening, workplace safety

## Abstract

Tuberculosis (TB) does not respect borders, and migration confounds global TB control and elimination. Systematic screening of immigrants from TB high burden settings and—to a lesser degree TB infection (TBI)—is recommended in most countries with a low incidence of TB. The aim of the study was to evaluate the views of a diverse group of international health professionals on TB management among migrants. Participants expressed their level of agreement using a six-point Likert scale with different statements in an online survey available in English, French, Mandarin, Spanish, Portuguese and Russian. The survey consisted of eight sections, covering TB and TBI screening and treatment in migrants. A total of 1055 respondents from 80 countries and territories participated between November 2019 and April 2020. The largest professional groups were pulmonologists (16.8%), other clinicians (30.4%), and nurses (11.8%). Participants generally supported infection control and TB surveillance established practices (administrative interventions, personal protection, etc.), while they disagreed on how to diagnose and manage both TB and TBI, particularly on which TBI regimens to use and when patients should be hospitalised. The results of this first knowledge, attitude and practice study on TB screening and treatment in migrants will inform public health policy and educational resources.

## 1. Introduction

Tuberculosis (TB) disease continues to be a global health crisis, with an estimated 25% of individuals globally affected by TB infection (TBI), over 10 million new active TB cases and 1.4 million deaths per year [1,2,3].

TB does not respect borders, and migration from high- to low incidence countries confounds global TB control and elimination [4,5,6,7]. In Europe, the proportion of TB cases in foreign-born versus native persons varies significantly among countries, with cases in migrants making up approximately one third of all TB cases in 2019 [8]. The incidence of TB among migrants is highest in the first years after arrival in the destination country [9,10].

Traditionally, TB control is aimed at rapidly identifying and treating infectious TB cases [11] and TB elimination is the strategy aimed at preventing future cases, including diagnosis and treatment of TBI [12,13,14,15,16]. Systematic screening of immigrants from TB high burden settings (by chest radiography—CXR) [17,18], and for TBI is suggested by the World Health Organization (WHO) using one of the two approved tests (tuberculin skin test [TST] or interferon gamma release assays [IGRAs]) [19,20,21]. The ECDC (European Centre for Disease Prevention and Control) recommends the use of CXR at arrival in the destination country and TST and/or IGRA for migrants from high-TB-incidence countries [22]. There is a wide variety of screening policies and programmes, in settings (pre- on, post-arrival), eligibility criteria and screening tools [5,23]. Recent guidelines by WHO [19,20] and the ECDC [22] make recommendations on TBI management strategies among migrants to reach the TB elimination targets set for Europe [4,10,24,25,26,27]. However, in practice a variety of strategies exist among different European countries to manage TB disease and infection [6].

Understanding the knowledge of local health staff which may influence TB control practices in different settings is important to improve the public health response. A global survey of TB screening and treatment among migrants is not available in the literature.

The aim of the present study is to investigate through a survey questionnaire related to TB and migration, including diagnosis and treatment of TB and TBI, to which extent a diverse group of international health professionals agree or disagree with the different statements proposed.

The results will inform educational resources on the topic.

## 2. Results

A total of 1055 respondents from 80 countries and territories participated in the survey. Of these, 234 answered in English, 135 in French, 20 in Mandarin, 196 in Portuguese, 311 in Russian and 159 in Spanish.

**Figure 1:**
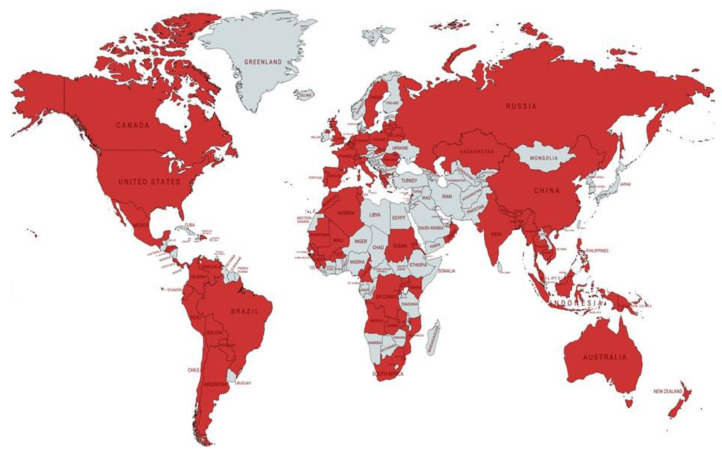
Countries participating (in red).

Figure 1 and Table 1 show the geographical location of the survey participants. The countries with the most responders (more than 10 participants each) were Argentina, Australia, Brazil, China, Ecuador, France, Italy, Lithuania, Mexico, Oman, Peru, the Russian Federation, Slovakia, Spain, Tunisia, and the USA. The majority of WHO high TB burden countries were represented [1].

**Figure 2:**
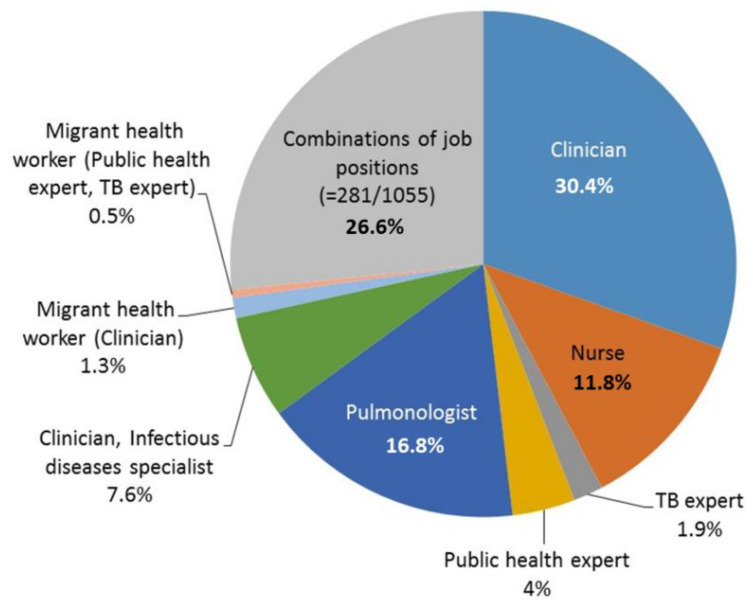
Profession of the 1055 respondents.

Figure 2 summarizes the participants’ working profile. The three top represented professional groups included medical doctors with expertise in respiratory medicine (16.8%) and different clinical areas (30.4%), while 11.8% of respondents were nurses.

**Figure 3:**
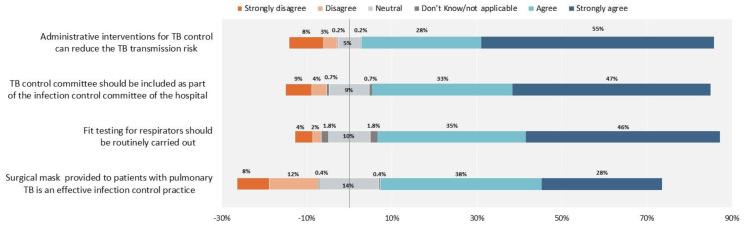
Infection control measures The proportion of healthcare workers who responded “Neutral” and “Don’t know/not applicable”, are reported 50% on the right side with agreement and 50% on the left side with disagreement.

**Figure 4:**
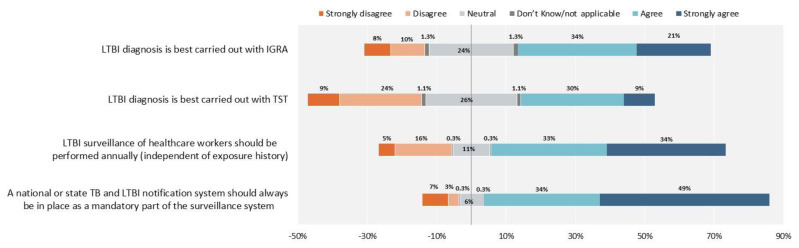
Latent tuberculosis infection diagnosis and surveillance. The proportion of healthcare workers who responded “Neutral” and “Don’t know/not applicable”, are reported 50% on the right side with agreement and 50% on the left side with disagreement.

**Figure 5:**
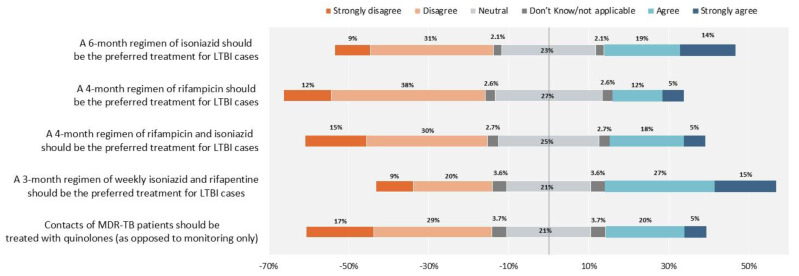
Latent tuberculosis infection treatment. The proportion of healthcare workers who responded “Neutral” and “Don’t know/not applicable”, are reported 50% on the right side with agreement and 50% on the left side with disagreement.

**Figure 6:**
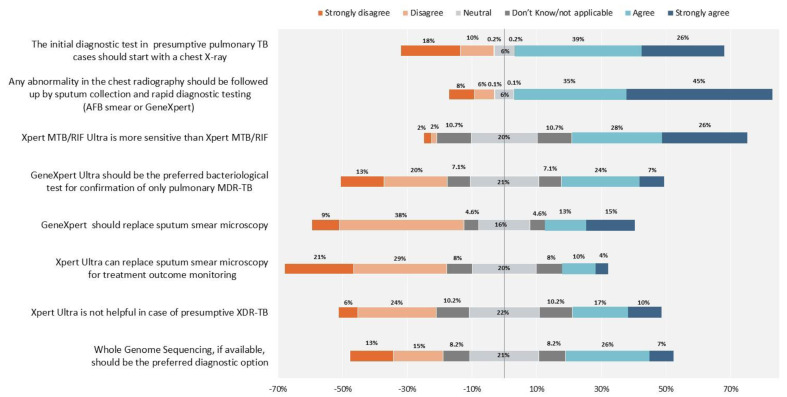
Tuberculosis diagnosis. The proportion of healthcare workers who responded “Neutral” and “Don’t know/not applicable”, are reported 50% on the right side with agreement and 50% on the left side with disagreement.

**Figure 7:**
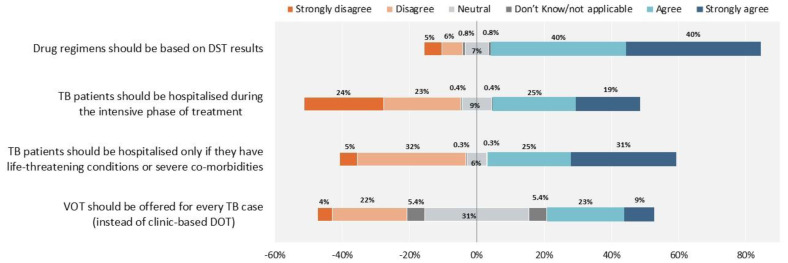
Tuberculosis treatment management. The proportion of healthcare workers who responded “Neutral” and “Don’t know/not applicable”, are reported 50% on the right side with agreement and 50% on the left side with disagreement.

**Figure 8:**
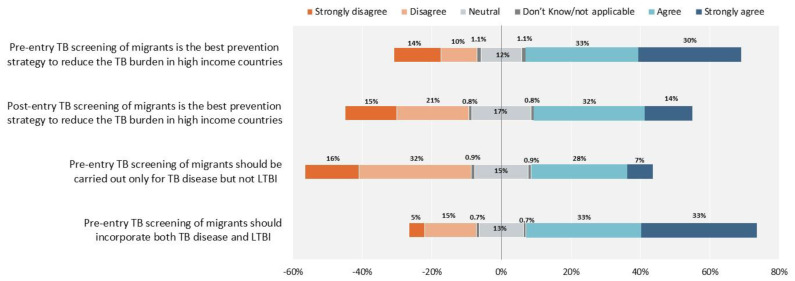
Tuberculosis screening. The proportion of healthcare workers who responded “Neutral” and “Don’t know/not applicable”, are reported 50% on the right side with agreement and 50% on the left side with disagreement.

The main results are summarized in Figure 3, Figure 4, Figure 5, Figure 6, Figure 7 and Figure 8. Figure 3 shows clear agreement of responses in supporting the importance of the standard elements of an infection control plan, e.g., administrative measures, implementation of an infection control committee at facility level, implementation of respirator fit testing for health staff to ensure effective individual protection and consistent use of surgical masks for patients.

Figure 4 reports that some disagreement exists about the ideal test to use to diagnose TB infection (TST or IGRAs), while agreement exists on the importance of implementing surveillance for TB infection (via a register) and of performing annual screening of health care workers for TB infection.

Figure 5 highlights the existing disagreement on what regimens should be used to treat TB infection. Respondents were neutral and disagreed regarding which regimens to use, including the ‘classic’ 6 month isoniazid regimen, the short 4-month rifampicin or 3-month isoniazid plus rifampicin regimens, and the newer 3-month rifapentine-based regimen. Furthermore, neutral and disagreement scores prevailed on the use of fluoroquinolone-based regimens to treat contacts of patients affected by multi-drug resistant tuberculosis (MDR-TB).

In Figure 6 agreement exists on the importance of performing CXR and bacteriological examinations in the case of abnormal CXR findings in individuals with presumptive TB. Participants reported conflicting statements on the performance and potential of Xpert MTB/RIF Ultra versus the ‘classic’ Xpert MTB/Rif. Furthermore, participants gave importance to performing Xpert at the clinical site, when available.

As reported in Figure 7, participants agreed on the importance of drug susceptibility testing (DST) in designing treatment regimens, while disagreeing on the need for routine hospitalization of patients. Furthermore, there is clear agreement on the importance of video-observed treatment (VOT).

Some disagreement also exists on the importance of pre- versus post-entry screening, and on the inclusion or not of TB infection screening (Figure 8).

## 3. Discussion

Our results showed that substantial agreement exists on several elements of the management strategy of TB in migrants among a diverse group of health professionals globally.

Most respondents strongly agreed on the importance of infection control measures although, as discussed below, full agreement does not exist on the recent WHO recommendation to reduce unnecessary hospitalization to lower transmission of *M. tuberculosis* [28,29]. This may result from a combination of national traditions and local regulations, often aiming at only discharging patients from hospital care after achieving bacteriological conversion [28,29].

In terms of staff protection, prevention and safety of working environments, there was strong agreement that health care workers (HCWs) should be screened annually for TB infection. While regular TB screening of HCWs is recommended in some contexts, a number of low incidence countries no longer recommend that HCWs are routinely tested for TB infection every year.

In the USA, the Centers for Disease Control and Prevention (CDC) and the National Tuberculosis Controllers Association (NTCA) abandoned their previous recommendation to perform routine serial TB testing of HCWs at specific intervals in their updated guidelines published in 2019 [30]. Occupational transmission of TB in healthcare settings has become rare in many low incidence settings, as the incidence of TB has declined over the years and infection control measures in health care settings have been successfully implemented. One of the largest cohort studies of more than 40,000 HCWs in the USA published in 2017 demonstrated an extremely low risk of occupational TB exposure among TST converters and no resulting TB cases [31]. The general support for regular TB screening among HCWs in our survey can likely be explained with the large proportion of study participants from higher incidence settings, and countries that currently use regular TB screening among HCWs.

The overwhelming majority of participants agreed on the importance of implementing a register-based surveillance of TBI to inform progress towards global TB elimination [19,20,21].

Most participants supported the use of DST to guide the choice of regimens for drug-resistant TB. This aligns with recommendations by WHO and the ATS (American Thoracic Society)/CDC (U.S. Centers for Disease Control and Prevention)/ERS (European Respiratory Society)/IDSA (Infectious Diseases Society of America) Clinical Practice Guidelines for the treatment of drug-resistant TB in low-incidence settings [3,32,33].

The main areas of disagreement among participants in our study related to the optimal test used to diagnose TB infection (TST or IGRAs) and the best regimen to treat it. The advantages and disadvantages of IGRAs and the TST have been widely debated [21]. Compared to the TST, IGRAs do not rely on the subjective reading of a skin induration by a HCW, only require a single patient visit, have generally better sensitivity and specificity, and are not impacted by previous BCG vaccination or exposure to non-tuberculous mycobacteria. On the other hand, IGRAs are more expensive than the TST and require special laboratory infrastructure and supplies.

Our survey participants had different opinions about the best treatment regimen for tuberculosis infection (TBI). Some participants preferred the ‘classical’ isoniazid-based regimen and others the new shorter regimens including 3 months of weekly isoniazid and rifapentine. The high cost of rifapentine, its adverse events and potential difficulties in procuring it may explain these findings. Participants disagreed on whether contacts of patients with MDR-TB should be treated with fluoroquinolones, as recently conditionally recommended by WHO and scientific societies [19,21,33]. This is an emerging field, and respondents may not be as familiar with emerging international thought and guidelines, skepticism towards guideline recommendations in the absence of evidence from randomized controlled trials, and concerns about increasing fluoroquinolone resistance in the community by using these drugs as a preventive treatment. The outcome of ongoing randomized trials on MDR-TB preventative therapy will be valuable for supporting greater consistency of practice in this area.

The survey participants did not prefer the Xpert Ultra over the basic Xpert test to diagnose TB disease in migrants, despite the Xpert Ultra having a higher sensitivity, and a requirement for less biological material for testing than the classic Xpert test. It is possible that participants were not always familiar with the differences between tests, especially as the Xpert test was just starting to be rolled-out in several countries at the time the survey was launched, or participants did not think that the potential advantages of the Xpert test were relevant when testing for active TB in migrants.

There was significant variation in what participants perceived to be the best timing to screen migrants, either before departure or after arrival. Some countries have implemented both pre entry screening—as a prerequisite for obtaining a visa—and migrant screening after arrival (applicable only for legal migrants), either in a dedicated TB center or with a general practitioner.

The variation in participants’ views on best screening practices is reflected in different approaches to TB screening among migrants in different low-incidence countries [27] such as France [34,35], Germany, and the UK [36,37]. Many low-incidence countries, including some Western European countries, the UK, the USA, Canada, Australia, and New Zealand, have implemented pre-migration TB screening for migrants from countries with a high incidence of TB [23]. Pre-migration screening in migrants’ countries of origin (pre-entry) or in the destination country (at-entry) aims to identify cases of TB disease. In some countries, notably in Canada and Australia, the pre-migration screening also helps to identify migrants at high risk of subsequently developing TB disease (based on chest radiography changes consistent with TBI but negative sputum microbiology results at the time of the initial screening [38]. These migrants at high risk of developing TB are then followed-up in dedicated programmes in the destination country [39]. Compared with other vulnerable groups, such as contacts of patients with TB and persons with immunocompromising conditions, these migrants have the highest risk of developing TB and might benefit from follow-up with TB services [40]. Reducing barriers for migrants to access health services in the destination country and providing culturally appropriate TB services are important components of migrant TB programmes [41].

Chest radiography is widely used as a screening test to assess migrants for active TB. However, the accuracy of chest radiography results depends on the skills of the individual reading the image, and there is inter-reader variability. The use of computer-aided programs based on reading chest radiographs could overcome this limitation [42], with the technology becoming increasingly reliable [17].

The risk of developing TB disease is highest in the first 2 to 5 years after arrival in migrants with TBI [5,23,43], providing a rationale for TB preventive treatment shortly after arrival [19,20]. As with TB disease, there is significant variation among low-incidence countries in the way they have implemented TBI screening and treatment [6,23,44]. Many low-incidence settings have their own national guidelines for TB screening and treatment among migrants, which might differ from WHO guidelines [17,18]. The cost-effectiveness of different programmes focusing on screening for TBI and/or TB remains unclear [45]. Further studies in this area should be part of the future research agenda in TB [46,47]. A recent European project has underlined the importance and feasibility of developing multi-country databases to gather evidence on TB and TBI in migrants [48].

Participants in our survey agreed that adequate health education, embedded within patient-centred services, ideally taking advantage of cultural mediators, is likely to increase acceptance of and adherence to TBI treatment, which is supported by the literature [19,20,23]. These measures might be even more important in asylum seekers and refugees who have a higher risk to develop TB than regular migrants [23].

The main strength of our study is its large sample size (exceeding 1000 participants) and the number of countries involved (80) with representation from all continents. By involving major international and national TB societies and several National TB Control Programmes in different regions for participant recruitment, the planned sample size target of 1000 participants was reached.

An additional strength is that the survey was available in six different languages, allowing participants to respond in a language they are familiar with, although it was not feasible to develop the questionnaire in languages like Swahili or Hindi.

Despite our efforts to recruit participants from Africa and China, these settings remained under-represented, while settings where Russian is spoken were over-represented.

Another potential limitation of the study may be related to the participants’ interpretations of the questions, in spite of the effort done to provide background translation and adhere to existing recommendations on questionnaire development.

Overall the study findings are likely to be representative and generalizable to the global TB community.

## 4. Materials and Methods

### 4.1. Study Design

The previously established Global Tuberculosis Network (GTN) structure was used to support and conduct this study. We set up a Steering Committee including international opinion leaders with specific expertise in TB and migration representing the main scientific societies, associations and groups active in the field. The same methodology used in previous studies coordinated by the GTN was applied [49,50,51].

The project included, as the first step, a Delphi process to develop a questionnaire aimed at obtaining opinions about optimal TB and TBI testing and treatment among migrants.

In the Delphi process, a panel of TB experts (recognized key opinion leaders from different language areas) were asked to assign a weight to each proposed element of the survey in terms of importance for TB and migration. The proposed elements were based on a literature review, covering the relevant areas of TB and TBI prevention, diagnosis and treatment. Experts were asked to assign a score to each question. A five-point Likert scale was used to determine the importance of including an element in the survey (5: high importance; 1: low importance). Within the panel (n = 39), 24 (61.5%) were TB clinicians, twelve (30.8%) TB public health specialists, 2 (5.1%) methodologists and one TB paediatrician.

All invited experts submitted a valid Delphi questionnaire. Each proposed element of the questionnaire was re-evaluated in three rounds of comments until consensus was reached about the elements to be included in the final survey.

A target of 1000 responders was empirically established, allowing us to address a range of healthcare workers and policy workers in each of the countries.

### 4.2. Questionnaire Translation, Distribution and Data Collection

The online survey was developed in English, French, Mandarin, Spanish, Portuguese and Russian. The survey was translated from English into the different languages by the respective mother-language members of the expert panel and backward translation was performed. The survey included a total of 25 statements, organised into 8 sections as follows: Part 1: General information about respondents; Part 2: TB Infection control; Part 3: TBI diagnosis and surveillance; Part 4: TBI treatment; Part 5: TB diagnosis; Part 6: TB treatment management; Part 7: TB Contact investigation; Part 8: Screening for TB and TBI and management issues in migrants. Survey questions are included as Supplementary File S1. Participants were asked to answer the questions based on what they consider should be done in an ideal setting (where all existing tests and procedures are available and there are no resource constraints) expressing to which degree they agree or disagree with the different statements proposed.

The survey was distributed globally between November 2019 and April 2020 via e-mail from a range of TB experts within and outside the GTN in language commonly spoken in the region, through the active support of different scientific societies and National TB programmes: ALAT (Asociación Latino-americana de Tórax), the Gulf Respiratory Society, the Mexican Respiratory Society, SEPAR (Spanish Society of Pneumology and Thoracic Surgery), SBPT (Sociedade Brasileira de Pneumologia e Tisiologia), SPLF (Société de pneumologie de langue française) the Russian TB Society, the Moscow Respiratory Society, the Australasian Tuberculosis Forum, and WAidid (World Association for Infectious Diseases and Immunological Disorders) among others.

Data were collected anonymously. Information on the participant’s country and professional activity in the field of TB and migration was included in the questionnaire. Participants were asked to express their agreement with statements in the questionnaire using a Likert scale including the following answers: strongly agree, agree, neutral, disagree, strongly disagree and I do not know/not applicable.

### 4.3. Data Analysis

Data were analysed using Stata 13 (StataCorp, College Station, TX, USA) and a descriptive analysis was performed. In interpreting the data, we considered agree/strongly agree as agreement and disagree/strongly disagree as disagreement while neutral and don’t know/not applicable, are reported 50% with agreement and 50% with disagreement as they cannot be considered neither agreement nor disagreement.

### 4.4. Ethical Aspects

According to current standard set by European Directive 2001/20/EC, and their adoption to national regulations (e.g., UK National Research Ethics Service (NRES), Governance Arrangements for Research Ethics Committees, paragraph 2.3.13), Research Ethics Committee review is not required for research involving healthcare staff recruited as research participants by virtue of their professional role.

Data were safely stored in the coordinating centre according to the best practices on data safety and protection.

## 5. Conclusions

This is the first global survey available to describe the agreement of health professionals on statements related to TB screening and treatment among migrants. The results provided important insights regarding views on the optimal management of TB and TBI in migrants from a global perspective. Understanding the areas of agreement and disagreement (particularly on diagnosis and treatment of TBI) are important to ensure that the different programmatic components of TB control and elimination programmes are adequately implemented. They will be useful to inform future research and educational activities.

## Figures and Tables

**Table 1:** Details of the respondents by country.

Country You Spend the Majority of Your Work Relating to TB		
Albania 1	Greece 7	Peru 21
Algeria 8	Guinea 1	Philippines 1
Angola 1	Guinea Bissau 1	Poland 1
Argentina 20	Honduras 1	Portugal 7
Australia 20	India 5	R. of Moldova 1
Bangladesh 2	Indonesia 3	Romania 2
Belarus 1	Italy 29	Russian Federation 297
Belgium 1	Kazakhstan 1	Rwanda 1
Bhutan 1	Kenya 1	Senegal 3
Bolivia 1	Latvia 1	Sierra Leone 1
Brazil 188	Lebanon 3	Slovakia 10
Burkina Faso 1	Lithuania 18	South Africa 4
Cambodia 2	Luxembourg 1	Spain 20
Cameroon 2	Malaysia 1	Sudan 5
Chile 7	Mali 1	Sweden 2
China 20	Mauritania 1	Switzerland 5
Colombia 4	Mexico 24	Thailand 1
Congo 2	Morocco 1	Timor-Este 1
Canada 2	Mozambique 3	Tunisia 13
Costa Rica 1	Myanmar 1	Uganda 1
Dominican Republic 3	Nepal 4	United Kingdom 2
Ecuador 53	Netherlands 8	USA 11
El Salvador 1	New Zealand 1	Vanuatu Islands 1
Eritrea 1	Oman 16	Venezuela 2
Eswatini 1	Panama 1	Vietnam 2
France 155	Papua New Guinea 1	Zambia 1
Germany 2	Paraguay 3	

## Data Availability

Data are stored in the coordinating centre and are available upon request.

## Supplementary Materials

The following are available online at https://www.mdpi.com/article/10.3390/antibiotics10111355/s1, File S1: Delphi questionnaire on TB and migration: Delphi questionnaire on TB and migration.
