# Supplementary material for: Screening for Tuberculosis in Migrants: A Survey by the Global Tuberculosis Network
Source: Antibiotics (Basel). 2021 Nov 5;10(11):1355. doi: 10.3390/antibiotics10111355 (PMC8615134; doi:10.3390/antibiotics10111355)
Supplement: Supplementary file 1 [file antibiotics-10-01355-s001.zip › antibiotics-1425997-supplementary.pdf]

Supplementary File S1. Delphi questionnaire on TB and migration

QUESTIONS ON TB AND MIGRATION
QUESTIONNAIRE submitted to 1055 survey participants

Legend of acronyms

TB tuberculosis
LTBI latent tuberculosis infection
IGRA interferon gamma release assay
TST tuberculin skin test
BCG bacille Calmette–Guérin
AFB acid-fast bacilli
DST drug-susceptibility testing
VOT video-observed treatment
DOT directly observed treatment
MDR-TB multidrug-resistant tuberculosis

Part 1: General information about respondent

COUNTRY you spend the majority of your work relating to TB

ARE YOU (more than 1 answer possible):

-Clinician
- Nurse
- Public health expert
-TB expert
- Pulmonologist
- Infectious diseases specialist
- Migrant health worker

TYPE OF HEALTH-CARE FACILITY WHERE YOU WORK (more than 1 answer possible)

<u>TB care facility:</u>

- *Inpatient*
- *Outpatient*

<u>Other health care facility:</u>

- *Inpatient*: a) acute b) long-term
- *Out-patient*

<u>Public health Unit</u>
<u>Community setting</u>
<u>Prison</u>
<u>Cross-border/refugees setting</u>

**Part 2: TB Infection control**	**Strongly agree**	**Agree**	**Neutral**	**Disagree**	**Strongly disagree**	**Don’t know/not applicable**
Administrative interventions for TB control can reduce the TB transmission risk						
TB control committee should be included as part of the infection control committee of the hospital						
Fit testing for respirators should be routinely carried out						
A surgical mask provided to patients with pulmonary TB is an effective infection control practice						
Negative pressure isolation room should be available in all health care facilities where TB patients are admitted routinely						
Air exchange practices should always undergo routine maintenance in the health care facility where TB patients are admitted						
TB specialists should always be involved in developing TB infection control policies						
Training courses on TB infection control for all healthcare workers should be performed routinely						

**Part 3: LTBI diagnosis and surveillance**	**Strongly agree**	**Agree**	**Neutral**	**Disagree**	**Strongly disagree**	**Don’t know/not applicable**
LTBI diagnosis is best carried out with IGRA						
LTBI diagnosis is best carried out with TST						
LTBI diagnosis is best carried out with initial TST, followed by IGRA if the TST is positive and there is a history of BCG vaccination						
TB surveillance of healthcare workers should be performed annually (independent of exposure history)						
LTBI surveillance of healthcare workers should be performed annually (independent of exposure history)						
LTBI surveillance of medical/health science students should be mandatory						
A national or state TB and LTBI notification system should always be in place as a mandatory part of the surveillance system						

**Part 4: LTBI treatment**	**Strongly agree**	**Agree**	**Neutral**	**Disagree**	**Strongly disagree**	**Don't know/not applicable**
A 6-month regimen of isoniazid should be the preferred treatment for LTBI cases						
A 4-month regimen of rifampicin should be the preferred treatment for LTBI cases						
A 4-month regimen of rifampicin and isoniazid should be the preferred treatment for LTBI cases						
A 3-month regimen of weekly isoniazid and rifapentine should be the preferred treatment for LTBI cases						
IGRA should be performed at the end of the LTBI regimen						
IGRA should be performed during the LTBI therapy						
Contacts of MDR-TB patients should be treated with quinolones (as opposed to monitoring only)						
DOT should be ensured for the 12-dose (3 months) isoniazid-rifapentine LTBI treatment regimen						

**Part 5: TB diagnosis**	**Strongly agree**	**Agree**	**Neutral**	**Disagree**	**Strongly disagree**	**Don’t know/not applicable**
TB triage (patient isolation, infection control measures with personal protection for staff, rapid access to diagnostics, etc) should always be carried out in patients with presumptive pulmonary TB						
The initial diagnostic test in presumptive pulmonary TB cases should start with a chest X-ray						
Assessment of presumptive pulmonary TB cases should include a chest Computerised Tomography						
Any abnormality in the chest radiography should be followed up by sputum collection and rapid diagnostic testing (AFB smear or GeneXpert)						
Xpert MTB/RIF Ultra is more sensitive than Xpert MTB/RIF						
Xpert Ultra should be the preferred bacteriological test for confirmation of pulmonary TB cases						
GeneXpert Ultra should be the preferred bacteriological test for confirmation of only pulmonary MDR-TB						
GeneXpert should always be carried out with or before culture examination						
GeneXpert should replace sputum smear microscopy						
GeneXpert should be carried out only for sputum smear negative cases						
GeneXpert should be carried out for all presumptive pulmonary TB cases						
GeneXpert can be carried out for all respiratory specimens (e.g., sputum, BAL)						
Xpert Ultra is reliable for the diagnosis of extra-pulmonary TB						

Xpert Ultra can replace sputum smear microscopy for treatment outcome monitoring						
Xpert Ultra can replace sputum culture for treatment outcome monitoring						
Xpert Ultra can only be adopted for treatment outcome monitoring when sputum smear microscopy is positive						
Xpert Ultra needs to be integrated into a diagnostic algorithm including also other genetic tests (e.g. Inno-LIPA)						
Xpert Ultra is not helpful in case of presumptive XDR-TB						
Whole Genome Sequencing, if available, should be is the preferred diagnostic option						
Xpert Ultra cannot be replaced by Whole Genome Sequencing						
Sputum smear microscopy should never be prescribed if Xpert is available						
DST should always be requested when a patient with presumptive TB is admitted						
DST should only be requested for first-line drugs initially						
DST should be performed for either first- or second-line drugs for every patient with presumptive TB						
DST should be performed for first- and second-line drugs for every patient with presumed TB in settings/countries with high MDR-TB setting						
Only phenotypic DST should be performed						
Both phenotypic and molecular DST should be performed for either first or second-line drugs						

DST should be performed only if there is delay in sputum smear conversion						
If DST is performed in patients because they have delayed sputum smear conversion, only molecular DST should be performed.						
If DST is performed in patients because they have delayed sputum smear conversion, only phenotypic DST should be performed						

**Part 6: TB treatment management**	**Strongly agree**	**Agree**	**Neutral**	**Disagree**	**Strongly disagree**	**Don't know/not applicable**
Drug regimens should be based on DST results						
TB patients should be hospitalised during the intensive phase of treatment						
TB patients should be hospitalised for the intensive and the continuation phase						
TB patients should be hospitalised only if they have life-threatening conditions or severe co-morbidities						
DOT should be ensured for every TB case						
VOT should be offered for every TB case (instead of clinic-based DOT)						
DOT should only be used for vulnerable groups						
Active drug safety monitoring and management of adverse events of anti-TB drugs (aDSM) should be always implemented						

**Part 7: TB Contact investigation**	**Strongly agree**	**Agree**	**Neutral**	**Disagree**	**Strongly disagree**	**Don’t know/not applicable**
Contact investigation should be always carried out in case of a confirmed pulmonary TB (on GeneXpert, sputum smear or culture)						
Contact investigation should always be carried out in case of a confirmed extra-pulmonary TB case						

Part 8: Screening for TB and LTBI and management issues on migrants	Strongly agree	Agree	Neutral	Disagree	Strongly disagree	Don’t know/not applicable
Pre-entry TB screening of migrants is the best prevention strategy to reduce the TB burden in high income countries						
Post-entry TB screening of migrants is the best prevention strategy to reduce the TB burden in high income countries						
Pre- and post-entry TB screening of migrants are the best prevention strategy to reduce the TB burden in high income countries						
Pre-entry TB screening of migrants should be carried out only for TB disease but not LTBI						
Pre-entry TB screening of migrants should be carried out for LTBI, but not active TB						
Pre-entry TB screening of migrants should incorporate both TB disease and LTBI						
Pre-entry TB screening should be linked to post-arrival follow-up						
Post-entry TB screening of migrants should be carried out only for TB disease						
Post-entry TB screening of migrants should be carried out only for LTBI						
Post-entry TB screening of migrants should be carried out for TB disease and LTBI						
The TB follow-up of migrants should be carried out for two years after entry						
The TB follow-up of migrants should be carried out for five years after entry						
Taking a medical history is the best means to screen migrants with presumptive TB disease						
Taking a medical history and molecular testing are the best means to screen migrants with presumptive TB disease						

Taking a medical history and chest radiography are the best means to screen migrants with presumptive TB disease						
TST is the best screening test for LTBI in migrants from countries with a high TB incidence						
IGRA is the best screening test for LTBI in migrants from countries with a high TB incidence						
TST and subsequent IGRA (if the TST is positive and the migrant has a history of BCG) is the best screening algorithm for LTBI in migrants from countries with a high TB incidence						
LTBI treatment should be offered to all migrants from countries with a high incidence of TB, with evidence of LTBI, regardless of time since arrival						
LTBI treatment should be offered to migrants with evidence of LTBI within five years of arrival						
A LTBI register should be implemented for migrants after their entry						
A supra-national register for migrants diagnosed with TB should be implemented to improve the cross-border treatment follow-up, assuring privacy and protection of personal data						
TB should never be considered a reason to prevent the entry in a country						
TB should never be considered a reason to justify deportation						
Individuals who are sputum smear positive should not travel by air						
